# Evidence for a conserved CCAP-signaling pathway controlling ecdysis in a hemimetabolous insect, *Rhodnius prolixus*

**DOI:** 10.3389/fnins.2013.00207

**Published:** 2013-11-05

**Authors:** DoHee Lee, Ian Orchard, Angela B. Lange

**Affiliations:** Department of Biology, University of Toronto MississaugaMississauga, ON, Canada

**Keywords:** Chagas disease, crustacean cardioactive peptide, G-protein coupled receptor, hemipteran, RNAi, CNS

## Abstract

A vital feature in the success of Ecdysozoa is their ability to shed their exoskeleton (a process called ecdysis) such that they can grow or change their morphology. In holometabolous insects, these behaviors are orchestrated by the sequential actions of neuropeptides, one of which is crustacean cardioactive peptide (CCAP). Little is known about the control of ecdysis in hemimetabolous insects. Here, we report that CCAP is essential for successful ecdysis in the hemimetabolous insect, *Rhodnius prolixus*; the vector of Chagas disease. The first indication of CCAP's involvement in ecdysis was the observation of decreased staining intensity of CCAP-containing neurons immediately following ecdysis, indicative of the release of CCAP. The critical importance of the CCAP signaling pathway was further demonstrated by knockdown (as determined by qPCR and immunohistochemistry) of the *CCAP* and *CCAPR* transcripts utilizing dsRNA. This technique reduced the staining intensity of CCAP-containing neurons, and knocked down the transcript levels by up to 92%, with lethal consequences to the insect. Insects with these transcripts knocked down had very high mortality (up to 84%), typically at the expected time of the ecdysis sequence, or had ecdysis extremely delayed. This is the first report of the susceptibility of *R. prolixus* to dsRNA knockdown of neuropeptide and receptor transcripts, and the data clearly demonstrates the conserved nature of the CCAP signaling pathway in ecdysis between holometabolous and hemimetabolous insects.

## Introduction

Groups of Ecdysozoa including Arthropoda, Nematoda, and Cephalorhyncha possess a hard cuticle. This exoskeleton serves as protection against injury and desiccation, as a barrier against microorganisms, and as a framework for the attachment of muscles; however, this exoskeleton has the potential to interrupt the growth of Ecdysozoa (Aguinaldo et al., 1997). Thus, Ecdysozoa shed their exoskeleton in order to grow or change their morphology. The process of shedding the cuticle, or ecdysis, involves innate behaviors that are exhibited in distinct phases. Ecdysis provides an ideal model to analyze or understand a behavior at the molecular and cellular level.

Ecdysis consists of three distinct stages: pre-ecdysis, ecdysis, and post-ecdysis. Contractions of specific skeletal muscles occur in each of the stages and are orchestrated by the combined actions of hormonal and neural components involving peptidergic signaling pathways (Truman, 2005; Žitňan et al., 2007). Direct and indirect evidence in arthropods suggest that the major components of these peptidergic signaling pathways are pre-ecdysis triggering hormone (PETH), ecdysis triggering hormone (ETH), eclosion hormone (EH), crustacean cardioactive peptide (CCAP), and bursicon (Ewer and Truman, 1996; Gammie and Truman, 1997; Žitňan et al., 1999; Park et al., 2003a,b; Clark et al., 2004; Kim et al., 2006a,b; Arakane et al., 2008; Roller et al., 2010). There is considerable evidence from Crustacea and holometabolous insects that CCAP is a neuropeptide that regulates some of the ecdysis behavior. For example, CCAP is released around the time of ecdysis and activates the ecdysis motor program by turning off pre-ecdysis in *Manduca sexta* (Gammie and Truman, 1997; Žitňan and Adams, 2000; Fuse and Truman, 2002). Ablation of neurons expressing CCAP leads to failure of pupal ecdysis in *Drosophila melanogaster* (Park et al., 2003a; Clark et al., 2004; Kim et al., 2006a) and recently using a genetic analysis approach it was shown that *Drosophila* lacking both CCAP and *pburs* (*partner of bursicon* gene) function expressed a more severe defect at pupation than flies lacking either hormone (Lahr et al., 2012). Other studies have shown that reducing CCAP and its receptor transcript levels by RNA interference (RNAi) in *Tribolium castaneum* leads to a failure in ecdysis (Arakane et al., 2008; Li et al., 2011). CCAP levels are also increased up to 30-fold or more than 100-fold in the haemolymph during ecdysis in a crab, *Carcinus maenas* and a crayfish, *Orconectes limosus*, respectively, and drop to basal levels after ecdysis (Phlippen et al., 2000). These studies suggest that CCAP is critical during ecdysis in Crustacea and holometabolous insects; however, little is known about hemimetabolous insects, and nothing is known for the important vector of Chagas disease, *Rhodnius prolixus*. This medically-important insect provides an ideal model for studies in growth and development, including ecdysis, since in the unfed condition each instar remains in a state of arrested development and gorging on a blood meal initiates all of the physiological and endocrinological events leading to the next instar. Thus, these events can be easily timed and therefore studied accurately.

Recently, we cloned and characterized the CCAP gene in *R. prolixus* and mapped the expression of *CCAP* to cells in the CNS, and not in other tissues (Lee and Lange, 2011; Lee et al., 2011). We have also cloned and characterized the *CCAP receptor gene* (*RhoprCCAPR*) and have shown that *RhoprCCAPR* is upregulated prior to ecdysis in the heart and plays a role in regulating heartbeat rate (Lee et al., 2013). In the current study, we have investigated the developmental expression pattern of both *RhoprCCAP* and *RhoprCCAPR* during ecdysis in the central nervous system (CNS) and peripheral tissues of *R. prolixus*. We observed a reduction in the staining intensity in cell bodies and processes of CCAP-like immunoreactivity (IR) immediately following ecdysis. Utilizing RNAi, we have successfully knocked down *RhoprCCAP* and *RhoprCCAPR* transcripts, confirmed by qPCR and peptide-staining levels. Finally, we demonstrate that RhoprCCAP is critically important in *R. prolixus* ecdysis since interrupting the RhoprCCAP signaling pathway using RNAi leads to high mortality at ecdysis or delayed ecdysis. This is the first study to confirm the involvement of CCAP in ecdysis in a hemimetabolous insect, and illustrates the potential of using *R. prolixus* as a model system for examining peptidergic signaling during ecdysis.

## Materials and methods

### Animals

Fourth instars *R. prolixus* were allowed to gorge on defibrinated rabbit blood (Hemostat Laboratories, Dixon, CA, USA; supplied by Cedarlane, Burlington, ON, Canada) and then held in an incubator at 28°C in a 16:8 h light/dark cycle. The humidity of the chamber was 30%.

### Ecdysis behavioral sequence

Ecdysis of fully gorged 4th instar *R. prolixus* was investigated during the photophase. From the day that the insects were fed, their behavior during the molting cycle was observed. Some insects from Day 8 post-fed were transferred during the photophase to a Petri dish that contained a filter paper on which ecdysis took place. Specific behaviors exhibited during pre-ecdysis, ecdysis, and post-ecdysis were observed and photographed (Cybershot, Sony).

### Quantitative real time PCR (qPCR) analyses

CNS, salivary gland, and hindgut of 4th or 5th instar *R. prolixus* were dissected in RNase free physiological saline and stored in RNA later solution (Ambion, Austin, TX). Tissues were dissected from *R. prolixus* 4th instar (Day 4–9 post-fed) and 5th instar (Day 1–6 post-ecdysis). Total RNA was isolated from tissues using the Trizol® reagent (Ambion, Austin, TX) and first-strand cDNA was synthesized using 100 ng of total RNA as previously described (Lee et al., 2013). The reaction was diluted 20-fold with nuclease free water and qPCR analyses were carried out on a CFX96Touch™ Real-Time PCR Detection System (Bio-Rad Laboratories Inc., Hercules, CA, USA) using the Ssofast™ EvaGreen supermix (Bio-Rad Laboratories Inc., Hercules, CA, USA). Gene specific primers for *RhoprCCAP*, *RhoprCCAPR*, and the reference genes (ribosomal protein 49, rp49, and beta-actin) (Table 1) were designed to amplify target fragments of similar size across all samples. The reference genes have been validated as stable targets and used previously (Lee et al., 2013). Each primer set was designed with one primer over an exon/exon boundary and the primer efficiency was determined for each target. The relative expression was determined following the ΔΔCt method (Livak and Schmittgen, 2001; Pfaffl, 2001) and fold-differences were normalized to both of the reference genes, RP49 and beta-actin. qPCRs were repeated for a total of three biological replicates with three technical replicates each and included a no template control and a no reverse-transcriptase control. The means of the biological replicates were calculated along with their standard errors. In the dsRNA knockdowns, CNS or peripheral tissues (salivary glands, hindgut, and pool of tissues including dorsal vessel, fat body, and trachea) were collected from 4th instars 5 days post-injection with dsRNA or no injection, and quantified as above. qPCRs were repeated for a total of four biological replicates with three technical replicates each that included a no template control and a no reverse-transcriptase control.

**Table 1:** **CCAP and its receptor primers in *Rhodnius prolixus***.

**Primers**	**Oligo sequence (5′ to 3′)**
qPCR_CCAP_For1	CTGCAAAAAAGGCTTTATTTTCC
qPCR_CCAP_Rev2	TCCCATAACTTCGCTTCAGAC
qPCR_CCAP_R_For1	GCTTAGCACTGGATAATGGACTG
qPCR_CCAP_R_Rev1	TCAATACGCTGATCAGTCCAACT
qPCR_RP48_For1	GTGAAACTCAGGAGAAATTGGC
qPCR_RP48_Rev1	GCATCATCAACATCTCTAATTCCTTG
qPCR_Actin_For1	AGAGAAAAGATGACGCAGATAATGT
qPCR_Actin_Rev1	CGGCCAAATCCAATCG

*Gene-specific primers (GSP) for real time PCR were designed for the developmental expression profile analysis*.

### Immunohistochemisty

Whole mount immunohistochemistry was performed to determine the level of CCAP in the CNS (brain, subesophageal ganglion, prothoracic ganglion, mesothoracic ganglionic mass) during developmental stages as described by Lee and Lange (2011) with slight modifications as described below. The CNS of 4th (6 days post-fed) and 5th instar (1 h and 6 days post-ecdysis) *R. prolixus* was dissected under physiological saline (NaCl, 150 mmol L^−1^, KCl, 8.6 mmol L^−1^, CaCl_2_, 2 mmol L^−1^, Glucose, 34 mmol L^−1^, NaHCO_3_, 4 mmol L^−1^, MgCl_2_, 8.5 mmol L^−1^, HEPES, 5 mmol L^−1^, pH 7.0). The tissues were fixed with 2% paraformaldehyde at 4°C overnight and processed for immunohistochemistry (Lee and Lange, 2011). CCAP like-IR was observed under a confocal microscope with LSM image browser software (Zeiss, Jena, Germany). The CNS of 4th instar *R. prolixus* 5 days post-injection (either with dsCCAP, dsARG, or saline) was also dissected and processed for immunohistochemistry. Pre-absorption of the CCAP antiserum with 10^−5^ M CCAP at 4°C for 24 h eliminated all staining indicating that the antiserum was specific for CCAP.

### Double-stranded RNA (dsRNA) synthesis

Two overlapping fragments of the DNA template for dsRNA synthesis (see Table 2) were prepared with PCR by conjugating the T7 RNA polymerase promoter to the 5′ end (5′-taatacgactcactatagggaga-3′) on *RhoprCCAP*, *RhoprCCAPR*, or ampicillin resistant gene (*ARG*) as previously described (Lee et al., 2013). The PCR products were used as template for double stranded RNA (dsRNA) synthesis using the T7 Ribomax Express RNAi System (Promega, Madison, WI, USA). After synthesis, the dsRNA was precipitated with isopropanol and eluted in DEPC-treated water. It was then quantified at 260 nm wavelength using nanodrop. The quality of the dsRNA products was verified by 1% agarose gel electrophoresis and kept at −80°C until use. Before injection, the dsRNA was resuspended with DEPC-treated water at a concentration of 2 μg/μl.

**Table 2:** **Primers for generating templates of dsCCAP, dsCCAPR or the ampicillin resistance gene (dsARG)**.

**RNAi constructs**	**Oligo sequence (5′ to 3′)**
**PRIMERS TO GENERATE dsCCAP**	
dsCCAP_For1	**TAATACGACTCACTATAGGGAGA**GACGACTTTGTACGATCATTTTG
dsCCAP_For2	**TAATACGACTCACTATAGGGAGA**CTGTTCTAACCGACGATGTATTC
dsCCAP_Rev1	**TAATACGACTCACTATAGGGAGA**AATAATGACTCCTTTTCCTGATGG
dsCCAP_Rev2	**TAATACGACTCACTATAGGGAGA**CGGTCTGACTGCGssCTG
**PRIMERS TO GENERATE dsCCAPR**	
dsCCAPR_For1	**TAATACGACTCACTATAGGGAGA**CTGGATAATGGACTGGGTTATAAG
dsCCAPR_For2	**TAATACGACTCACTATAGGGAGA**TATCTGGAGGATCACGGTTG
dsCCAPR_Rev1	**TAATACGACTCACTATAGGGAGA**GAATAGTGGCTCTGCGTAACG
dsCCAPR_Rev2	**TAATACGACTCACTATAGGGAGA**TACTGGATTAGCTGCTGAATTGAG
**PRIMERS TO GENERATE dsARG**	
dsARG_For1	**TAATACGACTCACTATAGGGAGA**ATGAGTATTCAACATTTCCGTGTC
dsARG_For2	**TAATACGACTCACTATAGGGAGA**CAACAGCGGTAAGATCCTTG
dsARG_Rev1	**TAATACGACTCACTATAGGGAGA**GGCACCTATCTCAGCGATC
dsARG_Rev2	**TAATACGACTCACTATAGGGAGA**AATAGTTTGCGCAACGTTG

*The bold in dsRNA sequence denotes the T7 promoter site*.

### dsRNA delivery

Fourth instar *R. prolixus* were anesthetized with CO_2_ for 10 s and 2 μg of dsRNA was injected into the thoracic/abdominal haemocoel at the base of the metathoracic legs using a 5 μl Hamilton syringe. Five differently treated groups, each consisting of 20–30 bugs were used in this experiment as follows: (1) dsCCAP injected, (2) dsCCAPR injected, (3) dsCCAP and dsCCAPR injected, (4) negative control dsARG injected, and (5) no injection. All bugs were left for 1 h at room temperature to recover and then placed into an incubator at 28°C on a 16:8 h light/dark cycle. Mortality, abnormal behavior, and ecdysis (timing and behavior) were monitored daily. This experiment was repeated 3 times.

## Results

### *R. prolixus* ecdysis behavioral sequence

**Figure 1:**
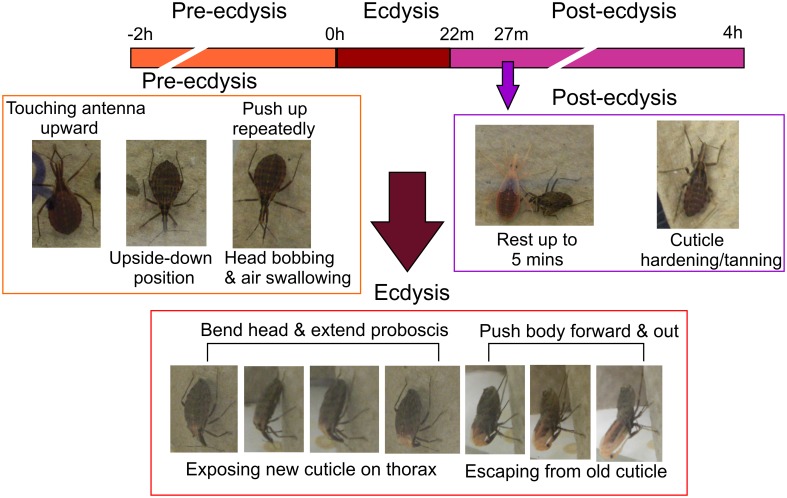
**Temporal sequence of pre-ecdysis, ecdysis, and post-ecdysis behaviors in 4th instar *Rhodnius prolixus*.** All characteristic behaviors during the different stages are boxed in different colors. Purple arrow denotes the end of the 5 min quiescent stage at the beginning of post-ecdysis. Observations based on 20 insects.

In 4th instar *R. prolixus* the ecdysis behavioral sequence commences on Day 9 post-feeding on defibrinated rabbit blood under our experimental conditions. Characteristic stages of the ecdysis behavioral sequence are observed, namely pre-ecdysis, ecdysis, and post-ecdysis as previously described (Ampleford and Steel, 1982a,b). Pre-ecdysis behavior occurs approximately 2 h prior to the ecdysis sequence (Figure 1). During that period *R. prolixus* are observed to repeatedly touch their antennae with their prothoracic legs and rub their legs against each other several times. They then invert themselves and remain vertically upside-down on filter paper for at least 2 h. As the timing of the ecdysis sequence approaches they bob their heads up and down, swallow air, and push up repeatedly using their forelegs. The ecdysis behavioral sequence in our regime (16L: 8D) occurs at 11 h after lights off, i.e., 3 h into the light. During the ecdysis sequence, *R. prolixus* bend their heads downward and in the middle of ecdysis, their proboscis extends with the tip pointing toward the abdomen (Figure 1). At this time, the new cuticle on the dorsal surface of the thorax and the first two abdominal segments is exposed due to longitudinal contraction of intersegmental skeletal muscles. During the last third of the ecdysis sequence, they are quiescent—a time devoted to shedding the cuticle from the delicate foregut and tracheae. Toward the end of the ecdysis sequence, all of the legs are free from the old cuticle and the hind legs are used to push the body forward and out of the old cuticle. The *R. prolixus* ecdysis sequence takes 22 ± 3 min (*n* = 20, Mean ± SE). After shedding the old cuticle, the insects remain close to their exuvia for up to 5 min. The new cuticle of the head, legs, and wing pads is very soft and light orange in color. Post-ecdysis behavior is observed after shedding the old cuticle. During this time the soft exoskeleton becomes darker and harder (Figure 1). Post-ecdysis behavior lasts for up to 4 h (*n* = 20).

### Developmental expression profile of *RhoprCCAP* and *RhoprCCAPR* transcript

**Figure 2:**
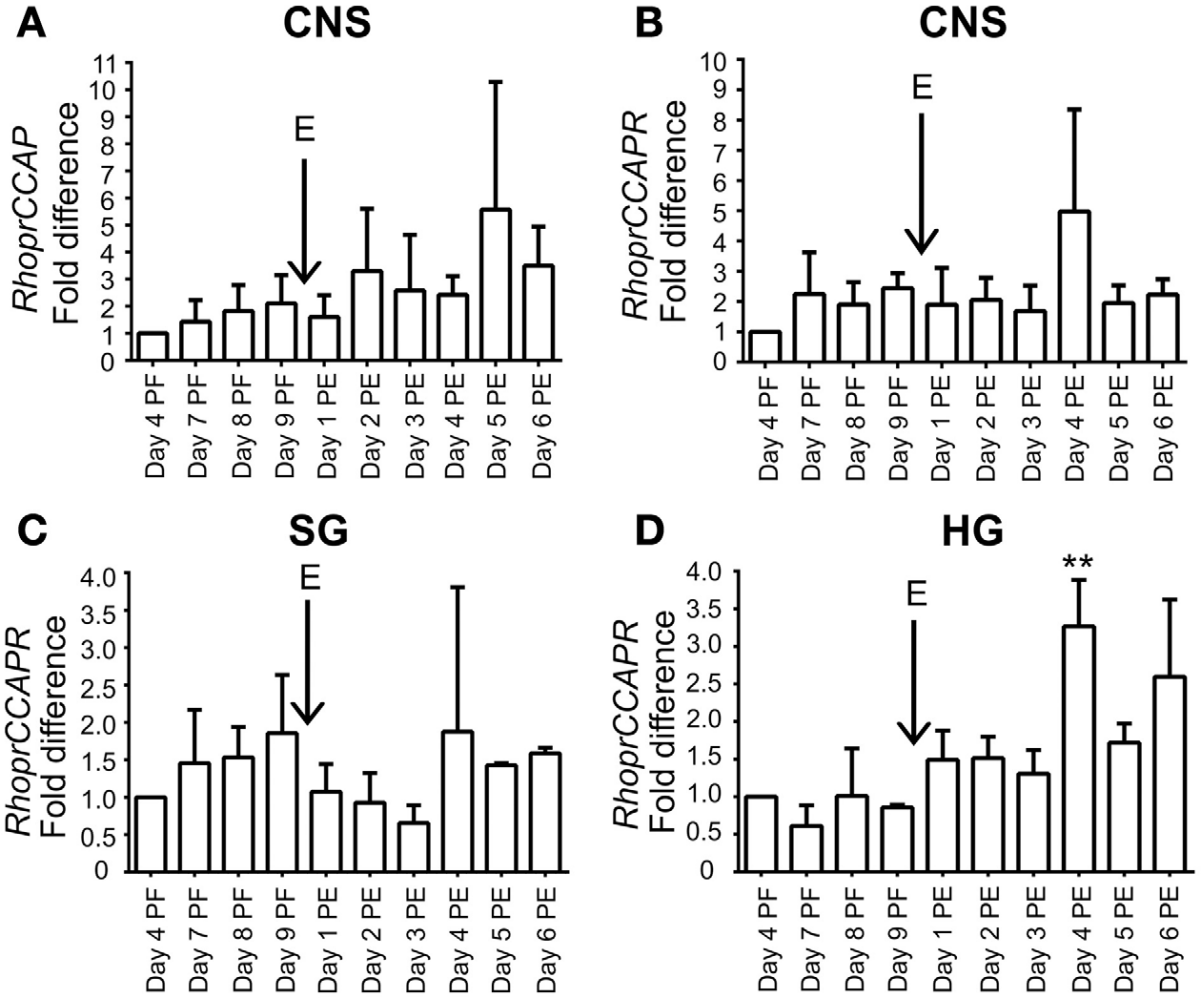
**Expression profile of *RhoprCCAP* and *RhoprCCAPR* transcript levels during development in different tissues of 4th and 5th instar *R. prolixus*. (A)** *RhoprCCAP* expression level in central nervous system (CNS). E represents day of ecdysis. **(B)** *RhoprCCAPR* transcript expression level in CNS. **(C)** *RhoprCCAPR* transcript expression in salivary gland (SG). **(D)** *RhoprCCAPR* transcript expression in hindgut (HG) was significantly increased Day 4 post-ecdysis (Day 4 PE) compared to Day 4 post-fed (Day 4 PF) as determined by ANOVA (*P* < 0.0004; Tukey's Test ^**^P < 0.01). Values are expressed as the mean ± s.e.m. (*n* = 3).

*RhoprCCAP* and *RhoprCCAPR* transcript expression is present throughout the days studied (Figure 2). In the hindgut, *RhoprCCAPR* transcript expression which was present on all days tested, was significantly (ANOVA *p* < 0.0004; Tukey's test *p* < 0.01) increased Day 4 post-ecdysis (Day 4 PE). (Figure 2).

### Developmental profile of *RhoprCCAP*

**Figure 3:**
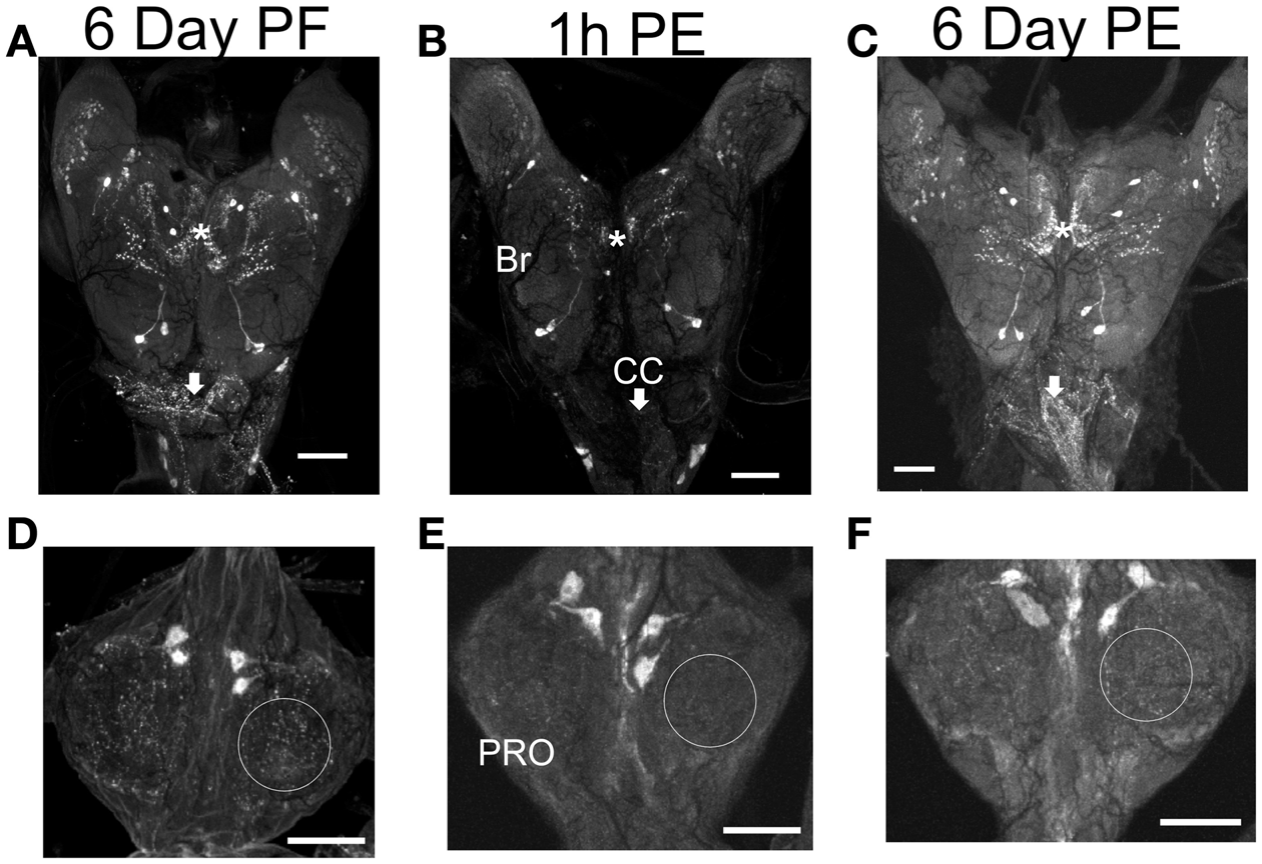
**CCAP-like immunoreactive staining in CNS.** Central nervous systems were dissected on Day 6 post-fed (PF) **(A,D)**, 1 h post-ecdysis (PE) **(B,E)** and Day 6 PE **(C,F)**. Reduction in staining intensity was observed in the neuropile and cell bodies in the brain (BR) (compare regions indicated by asterisks), processes in the corpus cardiacum (CC; compare regions indicated by arrows) and neuropile region in the prothoracic ganglion (PRO; compare regions indicated by circle) 1 h PE **(B,E)**. Staining intensity increased by Day 6 PE. *n* = 30/time point. Scale bars: 100 μm.

There is a substantial decrease in staining intensity of cell bodies and processes containing CCAP like-IR in the CNS 1 h post-ecdysis (PE) compared to Day 6 post-fed (PF) and Day 6 PE (Figure 3). In particular, CCAP like-IR in processes in the corpus cardiacum (CC) which forms an extensive neurohaemal plexus of terminal endings and varicosities were absent 1 h PE as were the extensive neuropilar regions in the brain and nerve cord.

### RNAi-mediated knockdown of *RhoprCCAP* and *RhoprCCAPR*

**Figure 4:**
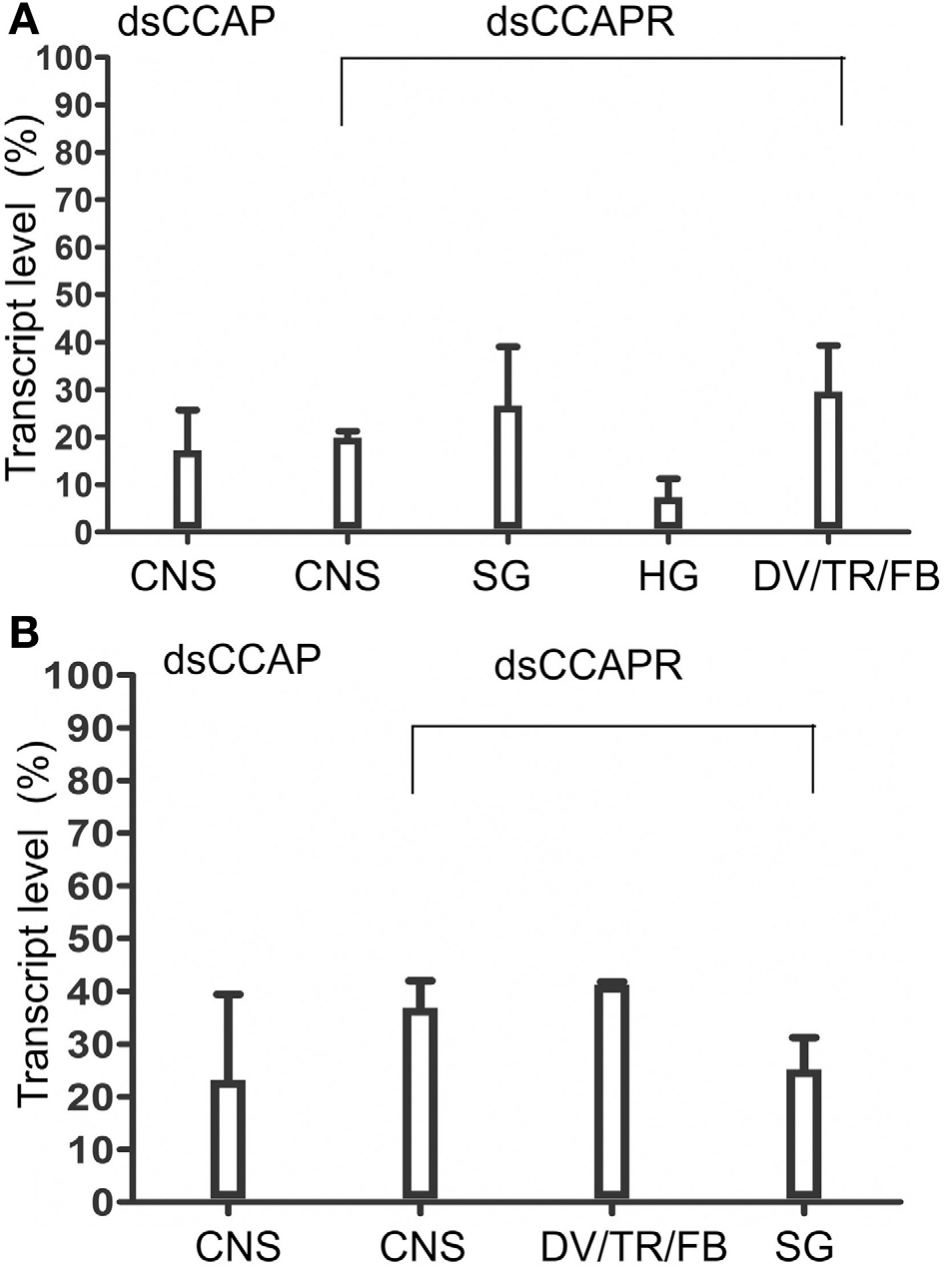
**Verification of dsRNA efficiency by real time qPCR. (A)** *RhoprCCAP* or *RhoprCCAPR* expression levels were reduced in 4th instar central nervous system (CNS), salivary gland (SG), hindgut (HG) and pools of tissues including dorsal vessel/trachea/fat body (DV/TR/FB) following injection of dsCCAP or dsCCAPR, relative to control dsARG injection. **(B)** *RhoprCCAP* and *RhoprCCAP* receptor expression levels were similarly reduced by injection of dsCCAP and dsCCAPR concurrently, compared with the control, dsARG. Values are expressed as the mean ± s.e.m. (*n* = 4).

**Figure 5:**
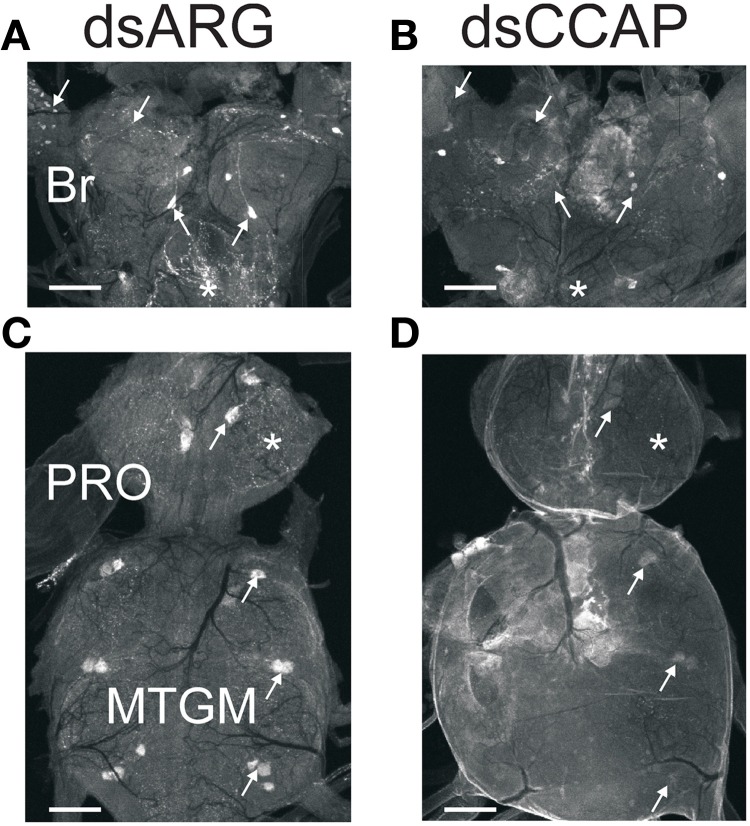
**Verification of dsRNA knockdown by immunohisto chemistry.** Central nervous systems from insects injected with dsARG or dsCCAP were examined Day 5 post-injection. **(A,C)** Controls (dsARG) display bright CCAP-like immunoreactive staining in cell bodies and neuropile processes (arrows). Asterisks denote staining in the corpus cardiacum **(A)** and neuropile **(C)**. **(B,D)** dsCCAP-treated insects were devoid of staining or had reduced staining intensity. Arrows indicate location of cell bodies and neuropile processes. Asterisks denote location of the corpus cardiacum **(B)** and neuropile **(D)**. Br, Brain; PRO, prothoracic ganglion; MTGM, mesothoracic ganglionic mass. *n* = 30/treatment. Scale bars: 100 μm.

Overall, *RhoprCCAP* and *RhoprCCAPR* transcript levels were dramatically reduced by 5 days after dsRNA injection (Day 9 PF) (Figure 4A). Control injections with dsARG did not reduce transcript levels relative to no injection (96 ± 3% of no injection). In the CNS, *RhoprCCAP* and *RhoprCCAPR* transcript levels were reduced by 82.6 ± 8.5% and 80.1 ± 1.3%, respectively. The percentage knockdown of the *RhoprCCAPR* transcript level was quantified in three peripheral tissues including the salivary glands, hindgut and a pool of tissues containing the dorsal vessel, trachea, and fat body and was reduced by 73.3 ± 12.4%, 92.7 ± 3.9%, and 70.4 ± 9.7%, respectively compared to the dsARG control group. In the double knockdown group (dsCCAP and dsCCAPR), the *RhoprCCAP* transcript level was decreased by 78 ± 18% in CNS, and the *RhoprCCAPR* transcript level was decreased in the CNS, pool of tissues containing the dorsal vessel, trachea, and fat body and the salivary glands with percentage knockdown of 63.1 ± 5.1%, 58.2 ± 0.2%, and 74.8 ± 6.0%, respectively (Figure 4B). dsRNA covering different regions of the same transcript was injected into *R. prolixus* and produced the same results, confirming the specificity of RNAi. Also, immunohistochemical analysis verified the reduction in peptide level caused by dsCCAP (Figure 5). Compared to the dsARG control group, the staining of cell bodies and processes containing CCAP-like IR was either absent or greatly reduced in the group injected with dsCCAP.

### The effects of disrupting the CCAP signaling pathway

#### Mortality

**Figure 6:**
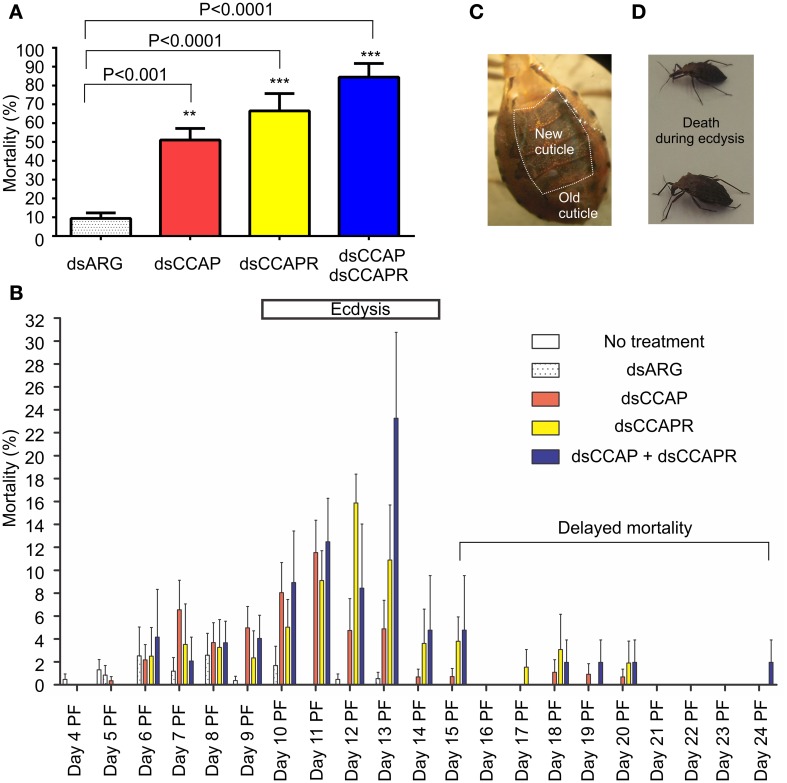
**Mortality increases in insects injected with dsRNA of RhoprCCAP, RhoprCCAPR or both in 4th instar *R. prolixus*.** Two micrograms of dsRNA for the ampicillin resistance gene (dsARG), the RhoprCCAP (dsCCAP), RhoprCCAPR (dsCCAPR), or both (dsCCAP and dsCCAPR) were injected into 4th instar *R. prolixus* on Day 4 post-fed (PF). **(A)** Insects injected with dsCCAP, dsCCAPR or dsCCAP and dsCCAPR have high mortality rate. Asterisks indicate significant differences compared to the control group that was injected with dsARG (unpaired *t*-test, ^**^P < 0.001; ^***^P < 0.0001). **(B)** Day on which mortality occurred. Bar denotes expected time of ecdysis. **(C)** Photo of a bug that died during the ecdysis sequence. The new cuticle has been laid down under the old cuticle. **(D)** Photo of two bugs that died after initiating the ecdysis sequence. Proboscis was extended posteriorly, body partially bent, but unable to complete ecdysis. Each group consisted of 20–30 bugs and this experiment was repeated 3 times. Values are expressed as the mean ± s.e.m. (*n* = 3).

*R. prolixus* injected with dsCCAP, dsCCAPR or both dsCCAP and dsCCAPR have high mortality when monitored over a time span of 24 days (Figure 6A). The mortality of insects injected with dsCCAP was 51.1 ± 6.1% and was significantly different from insects injected with dsARG as a control (unpaired *t*-test, *p* < 0.001). When dsCCAPR was injected, 66.5 ± 9.2% of the population died (unpaired *t*-test, *p* < 0.0001) and this was not significantly different from insects injected with dsCCAP (unpaired *t*-test, *p* = 0.084). Interestingly, when both *RhoprCCAP* and *RhoprCCAPR* transcripts were concurrently knocked down, the mortality rate increased to 84.4 ± 7.3% (unpaired *t*-test, *p* < 0.0001) and this knockdown was significantly higher than in those insects injected only with dsCCAP (unpaired *t*-test, *p* = 0.0069), but not with dsCCAPR (unpaired *t*-test, *p* = 0.1145).

Interestingly, in these RNAi experiments the majority of insects formed a new cuticle, in preparation for the ecdysis behavioral sequence, which was already separated from the old cuticle (Figure 6C). Overall, death of insects injected with dsCCAP and/or dsCCAPR occurred during the expected time of the ecdysis sequence (Day 9–14) (Figure 6B). In insects injected with dsCCAP, 47.6% of the population died between Day 5 and 14 PF while 3.4% of the population died from Day 15 to 20 (delayed mortality). In insects injected with dsCCAPR, 56.2% of the population died during Day 6–14 and 10.3% of the population died from Day 15 to 20. In insects injected with both dsCCAP and dsCCAPR, 71.8% of population died during Day 6–14, and 12.6% of them died from Day 15 to 24. None of the insects that died (even those with “delayed” mortality) had successfully completed the ecdysis sequence. Examination of the insects dying indicated that they died after initiating ecdysis behavior. Their proboscis was extended posteriorly and their body partially bent but they were unable to complete ecdysis and died in this position (Figure 6D).

#### Ecdysis

**Figure 7:**
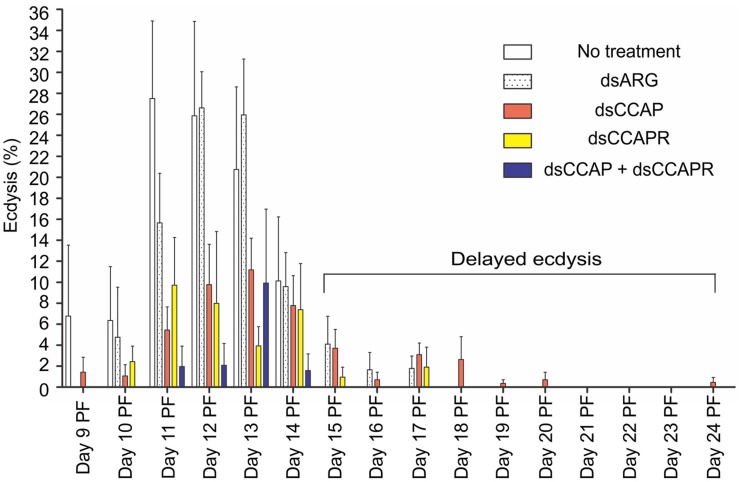
**Some 4th instar *R. prolixus* that had knockdown of *RhoprCCAP*, *RhoprCCAPR* or both did not die and were still able to ecdyse; however, many of them had delayed ecdysis.** Insects injected with dsCCAP, dsCCAPR, or dsCCAP and dsCCAPR displayed an ecdysis timing that was delayed (i.e., shifted to the right) compared to the control insects with some insects being delayed 24 days PF. Each group consisted of 20–30 bugs and this experiment was repeated 3 times. Values are expressed as the mean ± s.e.m. (*n* = 3).

In insects that received no injection, 97.4% of the population successfully completed the ecdysis behavioral sequence at the expected time between Days 9 and 14 PF (normal ecdysis) (Figure 7). A second set of control insects were injected with dsARG and in these 82.6% of the population successfully completed ecdysis with 7.5% showing a slightly delayed ecdysis (from Day 15 to 17 PF).

Although mortality was high for the experimental groups, some of the remaining insects injected with dsCCAP and/or dsCCAPR still were capable of normal ecdysis (36.7% of the dsCCAP injected insects; 31.4% injected with dsCCAPR; 15.6% injected with both dsCCAP and dsCCAPR) (Figure 7). Some of the insects injected with dsCCAP delayed their ecdysis behavioral sequence for up to Day 24 PF (11.7%) and 2.9% of insects injected with dsCCAPR also delayed their ecdysis for up to Day 17 PF.

**Figure 8:**
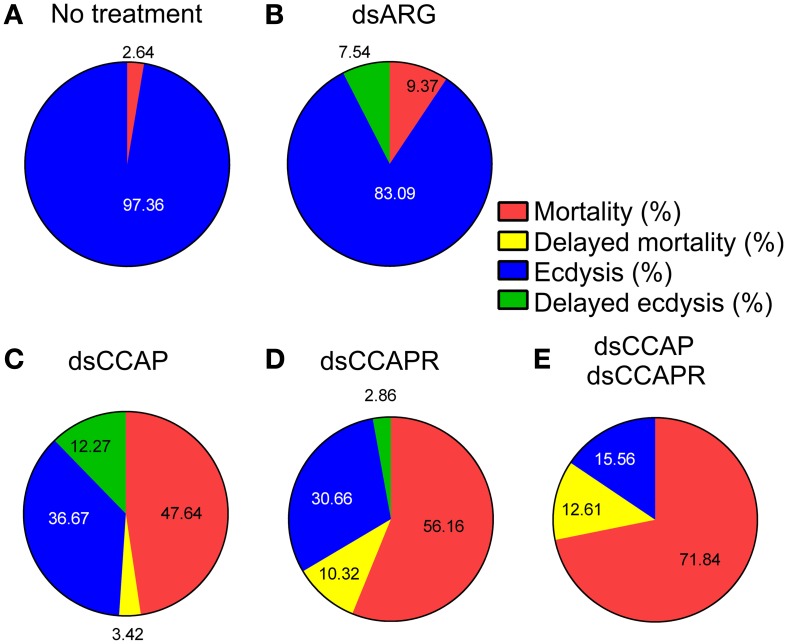
**Summary of the effects of knockdown of CCAP and CCAPR on *R. prolixus* ecdysis.** Four different categories were investigated; % mortality, % delayed mortality, % ecdysis and % delayed ecdysis. The percentage of mortality and delayed mortality was calculated up to Day 14 post-fed (PF), and after Day 15 PF, respectively. The percentage of ecdysis or delayed ecdysis was calculated on bugs up to Day 14 PF, and after Day15 PF, respectively. **(A)** No treatment. **(B)** Control injected with dsARG. **(C)** Injected with dsCCAP. **(D)** Injected with dsCCAPR. **(E)** Injected with both dsCCAP and dsCCAPR. Overall, high mortality rate and delayed ecdysis was observed in experimental groups that were treated with dsCCAP, dsCCAPR or both.

The data on mortality, delayed mortality, ecdysis, and delayed ecdysis are all summarized in Figure 8. Of the insects that ecdysed successfully, no defects or delays in cuticle hardening or tanning were observed.

## Discussion

Ecdysis is a complex process requiring a series of patterned behaviors and has been studied in detail in several holometabolous insects (see Žitňan and Adams, 2012). The ecdysis behavioral sequence is controlled by conserved neuronal networks and peptidergic signaling pathways and in holometabolous insects CCAP is one of the important neuropeptides involved in the ecdysis behavioral sequence (see Gammie and Truman, 1997; Fuse and Truman, 2002; Park et al., 2003a,b; Clark et al., 2004; Kim et al., 2006a,b; Arakane et al., 2008; Li et al., 2011; Žitňan and Adams, 2012). CCAP is co-expressed with other neuropeptides including bursicon, myoinhibiting peptides (MIPs), as well as ETH receptors (Kim et al., 2006a,b; Luan et al., 2006; Loveall and Deitcher, 2010).

In the hemimetabolous insect, *R. prolixus*, ecdysis timing was first studied by Ampleford and Steel (1982a,b). The ecdysis behavioral sequence occurs with a circadian rhythm, synchronized by lights off. *R. prolixus* demonstrates characteristic pre-ecdysis, ecdysis, and post-ecdysis behaviors in a similar fashion to holometabolous insects (see Gammie and Truman, 1997; Park et al., 2003a; Clark et al., 2004; Arakane et al., 2008). As might be expected for a peptide that is pleiotropic, the *CCAP* and *CCAPR* transcripts are present at all days tested, and CCAP is obviously involved in a variety of physiological processes. However, here we demonstrate that the CCAP signaling pathway is essential for successful ecdysis in *R. prolixus*; the first time that CCAP has been implicated in ecdysis in a hemimetabolous insect.

The RNAi technique has been used to investigate the functional roles of CCAP in ecdysis in this hemimetabolous insect, *R. prolixus*. Interfering with the CCAP signaling pathway disrupts ecdysis. Thus, injection of dsRNA into 4th instar *R. prolixus* resulted in a suppression of *RhoprCCAP* and *RhoprCCAPR* transcript levels as well as peptide content. *R. prolixus* with reduced transcript levels of *RhoprCCAP*, *RhoprCCAPR* or both have high mortality; the insects dying immediately prior to or during the ecdysis behavioral sequence. In a small number of cases the ecdysis behavioral sequence occurred but some were greatly delayed (up to 24 d). It is possible that in the insects that were successful at undergoing ecdysis or had delayed ecdysis, the CCAP signaling pathway had been disrupted but not to the same extent as in the insects that died during ecdysis (e.g., efficiency of knockdown). The involvement of CCAP in ecdysis is consistent with other studies. For example, the highest expression level of *CCAP* was observed during pre-moult and the lowest level was observed during post-molt in Crustacea (Chung et al., 2006). Also, peak *CCAP* receptor expression was observed during late pupal stages in *D. melanogaster* and *Anopheles gambiae* (Cazzamali et al., 2003; Belmont et al., 2006; Estevez-Lao et al., 2013). In *T. castaneum*, *CCAP* and its receptor transcript levels were highest during the mid-pupal stage but declined thereafter (Arakane et al., 2008). We observed a substantial reduction in the staining intensity of cell bodies and processes containing CCAP like-IR 1 h PE. Since we did not observe any decrease in CCAP transcript levels at these times, this decrease in staining is suggestive of CCAP being released from its stores (although we cannot discount that the stored CCAP is being enzymatically degraded). In *D. melanogaster* larval and pupal ecdysis, a reduction in the staining of CCAP-like IR in axons in the CNS was also observed (Park et al., 2003a; Clark et al., 2004).

The possible importance of CCAP in hemimetabolous insects has been suggested before. In *Locusta migratoria* nymphs, ecdysis behavior is associated with cGMP elevation in a set of CCAP-containing neurons (Truman et al., 1996), as it is in some other insects (Ewer and Truman, 1996; Žitňan et al., 2002; see Žitňan and Adams, 2012). Also, in *Schistocerca gregaria*, air swallowing—an important feature of ecdysis, is driven by the frontal ganglion, and rhythmic motor patterns are initiated from the frontal ganglion by application of ETH and CCAP (Zilberstein et al., 2006). Thus, CCAP in *R. prolixus* may well be acting on central neurons to initiate or modulate programmed motor output for specific sequences of muscle contraction associated with ecdysis, as has been shown in a variety of holometabolous insects (see Žitňan and Adams, 2012). CCAP and its receptor are also associated with the salivary glands and hindgut in *R. prolixus*. CCAP has direct myotropic actions on several insect visceral/skeletal muscles (Donini et al., 2001, 2002; Lange and Patel, 2005; Lee and Lange, 2011). It would be interesting to see if CCAP is important for modulating contractions of some of the muscles involved in ecdysis behavior. The associations of CCAP with salivary glands suggests that CCAP may be involved in the control of this tissue, but this is yet to be tested.

In addition to the need for fine control of central neurons and skeletal and visceral muscles, ecdysis also demands a regulation of cardiovascular functions which affects heartbeat performance and the redirection of haemolymph flow (Phlippen et al., 2000). It is well known that CCAP is a cardioaccelerator in arthropods (Tublitz and Evans, 1986; Lehman et al., 1993; Nichols et al., 1999; Lee and Lange, 2011). *D. melanogaster* heartbeat frequency is increased during the last 10 h of adult development and peaks at 1 h before the ecdysis behavioral sequence in the white stage (Baker et al., 1999). Previously, we reported that *RhoprCCAPR* transcript level was up-regulated 10-fold in a pool of tissues containing the heart (dorsal vessel), trachea and fat body prior to ecdysis and dropped to basal levels post-ecdysis (Lee et al., 2013). This indicates that the heart/dorsal vessel may have increased sensitivity to CCAP during the ecdysis behavioral sequence. Also, reducing the *RhoprCCAPR* transcript level leads to significantly decreased heartbeat frequency (31.1%) *in vivo* (Lee et al., 2013). In other insects, CCAP is co-released with bursicon and MIPs—neuropeptides which are also important for ecdysis (Kim et al., 2006a,b; Woodruff et al., 2008). CCAP may help to disperse these neuropeptides throughout the haemocoel during the ecdysis behavioral sequence because of its ability to increase heartbeat rate. In addition, interfering with these other physiological functions of CCAP (e.g., heart and muscle stimulatory actions) might be causes of early mortality or delayed ecdysis in some of the insects tested.

Overall, our RNAi experiments followed by behavioral analysis reveal the essential roles of CCAP in *R. prolixus* ecdysis and this represents the first hemimetabolous insect to be studied in this way. In addition, this work emphasizes the conserved nature of the CCAP signaling pathway throughout insects and furthermore confirms the advantages of using *R. prolixus* as a model in light of the ability to precisely time events associated with growth and development.

### Conflict of interest statement

The authors declare that the research was conducted in the absence of any commercial or financial relationships that could be construed as a potential conflict of interest.
